# Reliability of ultrasound measurements of quadriceps muscle thickness in critically ill patients

**DOI:** 10.1186/s12871-018-0647-9

**Published:** 2018-12-27

**Authors:** Emmanuel Pardo, Hanen El Behi, Priscilla Boizeau, Franck Verdonk, Corinne Alberti, Thomas Lescot

**Affiliations:** 1 Anesthesiology and Critical Care Department, Saint-Antoine Hospital, Assistance publique-hôpitaux de Paris, Paris, France; 2 AP-HP, Inserm, Université de Paris Diderot, Sorbonne Paris Cité, Hôpital Robert Debré, Unité d’épidémiologie clinique, CIC-EC 1426, Paris, France; 3 Sorbonne Universités, UPMC Université de Paris 06, Paris, France

**Keywords:** Muscle wasting, Ultrasonography, Intensive care unit, Protein, Quadriceps muscle

## Abstract

**Abstract:**

**Background:**

Muscle wasting in critically ill patients is associated with negative clinical outcomes. Ultrasound *quadriceps femoris* muscle assessment may constitute a convenient tool to evaluate muscle wasting. Nevertheless, its reliability remains uncertain. Our primary aim was to study the intra- and inter-observer reliability of this technique. Our secondary aim was to assess the evolution of the quadriceps muscle during the first 3 weeks after ICU admission and its possible association with nutritional intake.

**Methods:**

This observational study included patients expected to stay more than 7 days in the ICU. Ultrasound quadriceps muscle thickness was measured with a 12 MHz linear transducer, by two trained physicians, on D1, D3, D5, D7 and D21. Two measurements sites were evaluated: on the midpoint or on the two-thirds of the length between the anterior superior iliac spine and the upper border of the patella. Intra and inter-observer reliability was assessed by calculating the intra-class correlation coefficient (ICC).

**Results:**

A total of 280 ultrasound quadriceps thickness measurements were performed on 29 critically ill patients. Intra-observer reliability’s ICC was 0.74 [95% CI 0.63; 0.84] at the “midpoint” site and 0.83 [95% CI 0.75; 0.9] at the “two-thirds” site. Inter-observer reliability’s ICC was 0.76 [95% CI, 0.66; 0.86] at the “midpoint” site and 0.81 [95% CI, 0.7; 0.9] at the “two-thirds” site. *Quadriceps femoris* muscle thickness decreased over 16% within the first week after ICU admission. No correlation was found between muscle loss and caloric (*p* = 0.96) or protein (*p* = 0.80) debt over the first week.

**Conclusion:**

The assessment by ultrasonography of the quadriceps muscle thickness reveals good intra- and inter-observer reliability and may constitute a promising tool to evaluate the effect of nutritional-based interventions on muscle wasting in critically ill patients.

**Trial registration:**

“Committee for the Protection of Human Subjects in Biomedical Research” - Paris Ile de France VI Pitié-Salpêtrière – 10/07/2014.

French Data Protection Committee (“Commission Nationale Informatique et Libertés”) - #1771144.

## Background

Muscle wasting (MW) occurs early and rapidly during the intensive care unit (ICU) stay and contributes significantly to the development of ICU acquired weakness described in 50 to 100% of critically ill survivors [1]. In critically ill patients, Puthucheary et al. reported a 12.5% loss of *rectus femoris* muscle cross-sectional area (CSA) 7 days after ICU admission, ascribed to an increased protein turnover and an imbalance between muscle protein synthesis and protein degradation [2–4]. From a conceptual point of view, muscle mass assessment in critically ill patients may help to detect at-risk patients and predict the patient outcome given that low skeletal muscle mass in critically ill patients has been independently associated with prolonged mechanical ventilation, prolonged ICU and hospital length of stay and mortality [5]. Furthermore, monitoring muscle mass during the ICU stay may allow physicians to successfully identify patients who would benefit the most from tailored nutritional interventions. Nevertheless, assessing body composition and more precisely lean mass remains challenging in this setting: different methods have been described and validated, but none of them can be applied in the ICU setting [6]. Anthropometric measurements such as tricipital skin-fold thickness and mid-arm muscle circumference may underestimate sarcopenia in critically ill patients considering the high incidence of subcutaneous edema [7]. Bioelectrical impedance is also subject to bias as it is linked to hydration status which makes it unreliable in the ICU [8]. Computed tomography [5], dual-energy X-ray absorptiometry [9] and magnetic resonance imaging [10] offer accurate estimations of muscle mass by analyzing a cross-section usually going through the third lumbar vertebrae. However, these imaging techniques are not compatible with bedside evaluation and require unnecessary radiations and perilous transports.

In the last decade, ultrasonography (US) has taken an increasing part in ICU daily patient management and has recently been suggested to measure muscle volume and architecture [11, 12]. The estimations revealed good accuracy compared with reference methods [13]. Therefore, US assessment may constitute a promising non-invasive bedside tool to measure muscle thickness instantaneously and repeatedly. Quadriceps muscle combined thickness evaluation is considered relevant because it has been described as a reliable reflection of muscle strength [11, 14]. Parameters obtained with ultrasound measurements could help physicians to dynamically monitor lean mass evolution across the ICU stay and consequently adapt nutritional support [15].

Existing data indicate acceptable reproducibility in healthy subjects; however only few data have been reported in critically ill patients [16]. US is known to be operator-dependent and measurements may be biased by variability in muscle compression, in site selection or in image analysis [17]. Two main measurement sites have been described in the literature: either at midpoint or at two-thirds of the length between the anterior superior iliac spine (ASIS) and the upper pole of the patella. No consensus has been made over the optimal protocol to assess quadriceps femoris muscle mass. Our main hypothesis is that US is a reliable tool to assess muscle wasting in critically ill patients.

The primary objective of this study is to assess the overall intra- and inter-observer reliability of quadriceps muscle thickness measured with ultrasound in a general population of critically ill patients; and more specifically, in two distinct measurement sites. The secondary objective is to describe the quadriceps muscle thickness evolution over the three first ICU weeks after admission and its association with nutritional intake.

## Methods

### Study design and population

This observational monocentric study was conducted in the surgical critical care unit of Saint-Antoine University Hospital in Paris, France and complied with published STROBE (Strengthening the Reporting of Observational Studies in Epidemiology) guidelines [18]. The access to health information was approved by an ethics committee (Committee for the Protection of “Human Subjects in Biomedical Research” - Paris Ile de France VI Pitié-Salpêtrière – 10/07/2014) and by the French Data Protection Committee (“*Commission Nationale Informatique et Libertés*” - #1771144) who waived the need for individual consent according to the French law at the time of the study.

We included patients (1) over 18 years old admitted to the ICU, (2) expected to stay more than 7 days and (3) who received a muscular ultrasound evaluation as part of their usual care. Were excluded from the study patients (1) with pre-existing neuromuscular pathology, (2) with a lower-limb amputation, (3) whose ultrasound data were missing or incomplete, and (4) patients discharged from the ICU before day 7.

### Image acquisition and measurement method

**Fig. 1:**
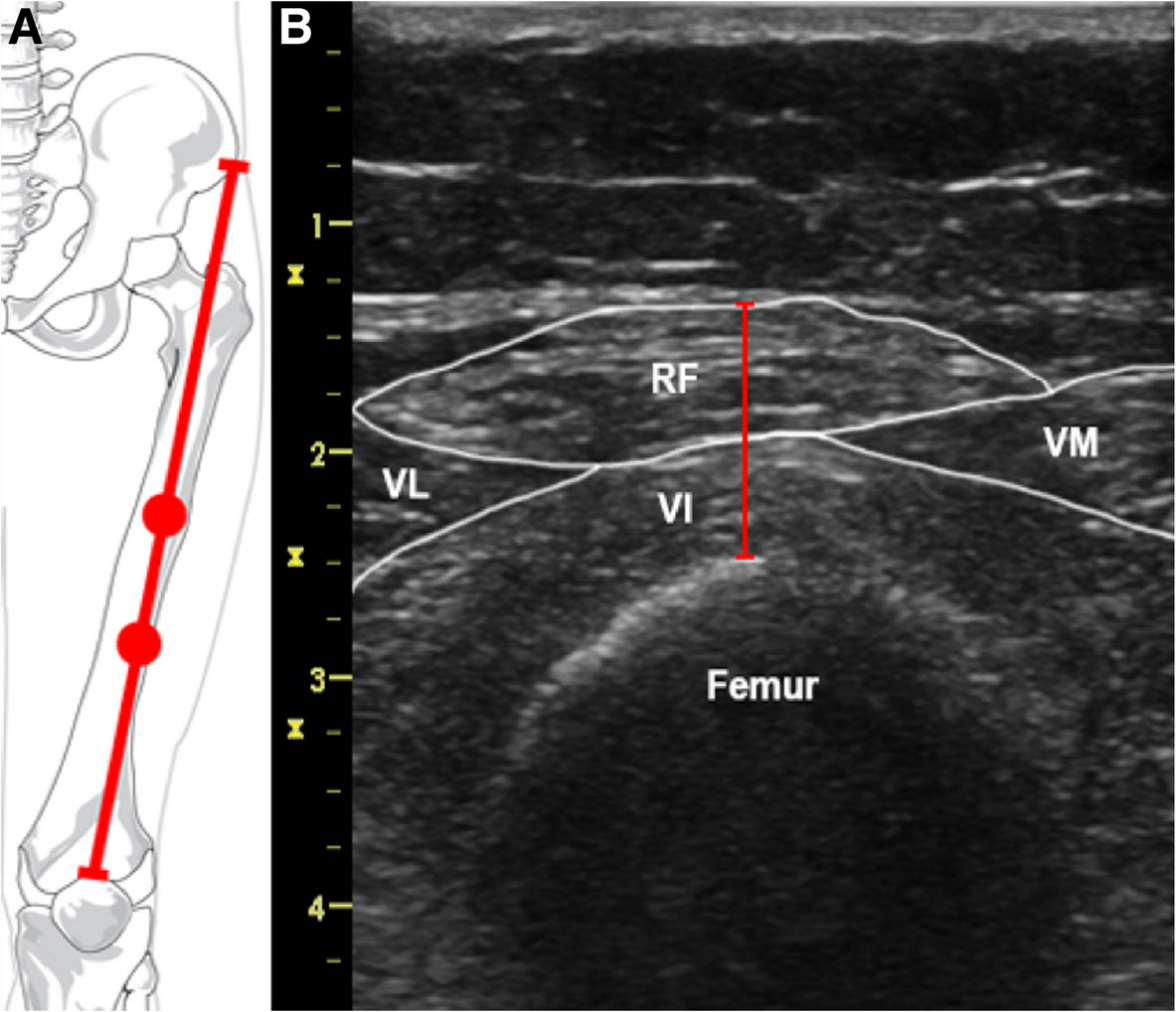
Ultrasound assessment of the *quadriceps femoris* muscle thickness. **a** Anatomical diagram locating the “midpoint” and the “two-thirds” measurement sites. From *Wikimedia Commons*. **b** Transverse ultrasound section made by linear probe at the midpoint site. RF: *rectus femoris*; VL: *vastus lateralis*; VM: *vastus medialis*; VI: *vastus intermedius*

Ultrasound measurement of quadriceps muscle thickness was performed as part of the usual clinical patient care developed within the unit. Measurements were realized according to the protocol described by Tillquist et al. [19] using a 12 MHz linear transducer connected to an ultrasound machine (Vivid i, GE Healthcare, United States). The ultrasound probe was placed perpendicular to the long axis of the thigh on its anterior surface, at the two-thirds (“two-thirds” site) and the midpoint (“midpoint” site) of the length between the anterior superior iliac spine (ASIS) and the upper border of the patella. Both measurement sites were determined using a measuring tape and analyzed in order to find the most reliable point. The obtained cross-section allowed the operator to visualize the *quadriceps femoris* muscle (*vastus medialis*, *vastus lateralis*, *vastus intermedius*, *rectus femoris*), the subcutaneous tissues, the adipose tissue and the femur. After identifying the muscle tissue, the thickness of the quadriceps muscle was obtained by measuring the distance between the cortex of the femur and the most superficial muscular fascia (Fig. 1). Measurements were performed by applying maximal compression on the ultrasound probe without inflicting pain, in order to prevent the underestimation of muscle wasting linked to subcutaneous edema. The effectiveness of the technique has been previously demonstrated in edematous patients [20]. For every measurement, the leg was maintained into a lower limb orthosis in order to keep the same exact position. As part of usual patient care in the unit protocol, measurements were made on both legs and on both sites repeatedly on D1, D3, D5, D7 and D21 of the ICU stay. In a quality approach purpose, additional measurements were performed to assess the agreement between values recorded by the same observer or by two different observers. Two trained operators (HEB and TL) carried out the measurements. For intra-observer reliability, the operator completed two consecutive measurements blindly without knowing the result of the first measurement. For inter-observer reliability, the two observers were unaware of each other’s results and rater order was allocated at random. Given the rapid occurrence of fluid shifts in critically ill patients, the time interval between measurements did not exceed 1 hour and measurements were not carried out during CRRT.

### Data collection

For this observational study, were collected general patient characteristics, ICU outcomes, risk factors associated with MW previously reported in the literature [21] and nutritional data: gender, weight, height, Body Mass Index (BMI), simplified acute physiology score II (SAPS II) score, length of the ICU stay, duration of mechanical ventilation, duration of renal replacement therapy, vasopressor administration, neuromuscular blocking agent administration, sedation time, daily caloric intake (expressed in kcal/day), daily protein intake (expressed in g/kg/day) and the assessment by US of the *quadriceps femoris* muscle thickness conducted by two observers. The muscle loss during the first week was obtained by calculating the difference between measurements made on D7 and D1. The cumulative nutritional debt during the first week corresponds to the difference between the recommended protein and caloric target according to international guidelines [22, 23] (1.3 g/kg/day; 25 kcal/kg/day) and the total caloric intake actually received.

### Statistical analysis

Qualitative variables are described as values and percentages; quantitative variables as medians [95% confidence interval]. Comparisons were made using non-parametric tests (Wilcoxon matched-pairs signed rank for quantitative variables). A *p* value of less than 0.05 was considered to indicate statistical significance. For each patient, muscle loss during the first 3 weeks was assessed by selecting the mean loss of the left and right *quadriceps femoris* combined thickness evaluated by the same observer (HEB) at the midpoint site. Non-parametric correlation analysis was completed using Spearman correlation test. The agreement between ultrasound measurements was represented by Bland and Altman plots, illustrating the difference between the two measurements of the same patient to the average of these two values. Good measurement reproducibility would generate a cloud of points close to the X axis regardless of the mean of the measurement pairs. Reliability between different measurements was assessed by Intraclass Correlation Coefficient (ICC). The ICCs were determined from the left and right thigh measurements and independently from laterality, and were interpreted as follows [24]: < 0: Poor agreement, 0.01–0.20: slight agreement, 0.21–0.40: fair agreement, 0.41–0.60: moderate agreement, 0.61–0.80: substantial agreement, 0.81–1.00: almost perfect agreement.

## Results

### Study demographics

Between December 2013 and April 2014, a total of 280 ultrasound quadriceps thickness measures were performed on the 29 patients included in the study. Characteristics of these patients are presented in Table 1. Median age was 64 years old [95% CI, 60–69] and median SAPS II score was 49 which corresponds to an expected mortality of 44%. Average length of stay was 14 days. Regarding patient care previously associated with MW, 79% of patients were mechanically ventilated, 62% were under continuous sedation, 17% received continuous neuromuscular-blocking agent and 41% were treated by vasopressors.

**Table 1:** Study demographics. Qualitative variables are described as values and percentages (%); quantitative variables as medians [95% CI]

	*N* = 29
Age (years)	64 [60–69]
Body mass index (kg/m^2^)	24 [22–27]
SAPS II	49 [42–56]
Vasopressors	12 (41)
Mechanical ventilation	23 (79)
Renal replacement therapy	8 (28)
Continuous sedation	18 (62)
Continuous Neuromuscular-blocking agent	5 (17)
Total energy daily intake D1-D7 (kcal)	1023 [508.1–1467]
Energy daily intake D1-D7 (kcal/kg)	14.6 [7.4–20.4]
Total Protein daily intake (g)	29.3 [8.7–52]
Protein daily intake (g/kg)	0.4 [0.3–0.7]
ICU Length of stay (days)	14 [13–28]

### Quadriceps femoris thickness measure reliability

#### Intra-observer reproducibility

**Fig. 2:**
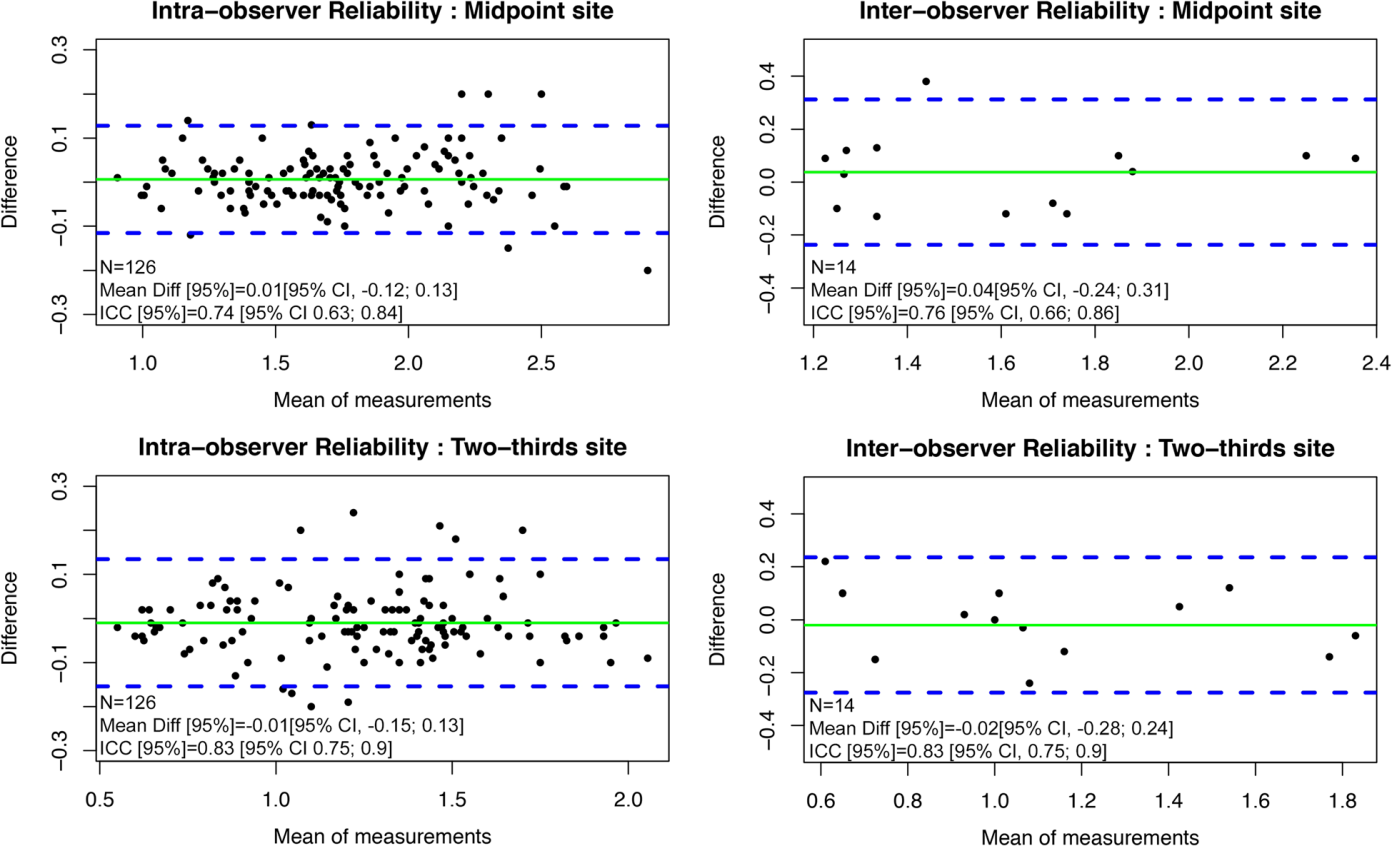
Intra-observer and inter-observer reliability independently of laterality at the two measurement sites. Bland Altman representation with mean differences (green solid line) and 1.96SD limits (blue dashed lines)

Sixty-three pairs of measurements were carried out for each lower limb and at each site (“midpoint” or “two-thirds”). Regarding the “midpoint” site, considering all the measurements (*n* = 126), independent from laterality, the mean difference between the two measurements was 0.01 cm [95% CI, − 0.12; 0.13] and the calculated ICC was 0.74 [95% CI 0.63; 0.84]. Regarding the “two-thirds” site, the average difference between the two measurements was − 0.01 cm [n = 126, 95% CI, − 0.15; 0.13] and the ICC was 0.83 [95% CI 0.75; 0.9] (Fig. 2).

#### Inter-observer reproducibility

Fourteen pairs of measurements were made blindly by two observers at each site. Concerning the “midpoint” site, the median difference between the two measurements was 0.04 cm [95% CI, − 0.24; 0.31] and the ICC was 0.76 [95% CI, 0.66; 0.86]. Concerning the “two-thirds” site, the median difference between the two measurements was − 0.02 cm [95% CI, − 0.28; 0.24] and the ICC was 0.83 [95% CI, 0.75; 0.9] (Fig. 2**).**

### Muscle thickness evolution

Quadriceps femoris thickness decreased promptly during the first week of the ICU stay.

**Fig. 3:**
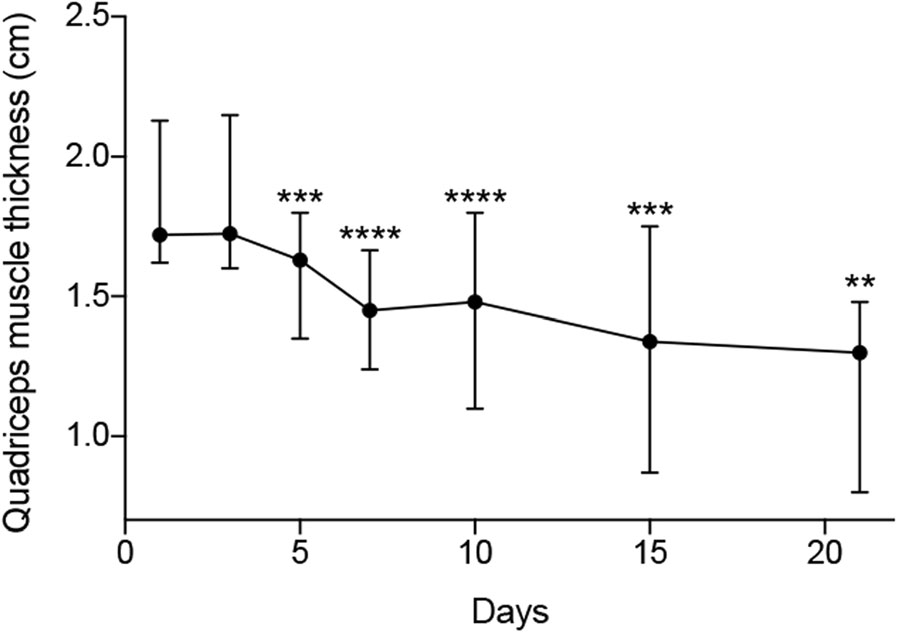
Evolution of *Quadriceps femoris* muscle thickness between D1 and D21. *P < 0.05; **P < 0.01; ***P < 0.001; ****P < 0.0001 (comparison with D1 assessment). Values are represented by medians and 95% confidence interval

Muscle mass loss was strongly correlated between the two legs with a *r* coefficient of 0.96 (*p* < 0.01) at midpoint and 0.93 (*p* < 0.01) at the two-thirds site. Median thickness at admission was 1.72 cm [95% CI, 1.62; 2.13] and decreased to 1.45 cm at D7 [95% CI, 1.24; 1.665], corresponding to a 16% significant variation in muscle thickness (− 0.32 cm [95% CI, − 0.43; − 0.2], *p* < 0.01). Median thickness decreased until 1.3 cm at D21 [95% CI, 0.80; 1.48] which corresponds to a total loss of 24% of muscle thickness (− 0.6 cm, [95% CI,-0.76; − 0.42], *p* < 0.01). Figure 3 depicts the evolution of quadriceps femoris muscle thickness between D1 and D21.

### Nutritional intake and muscle wasting

**Fig. 4:**
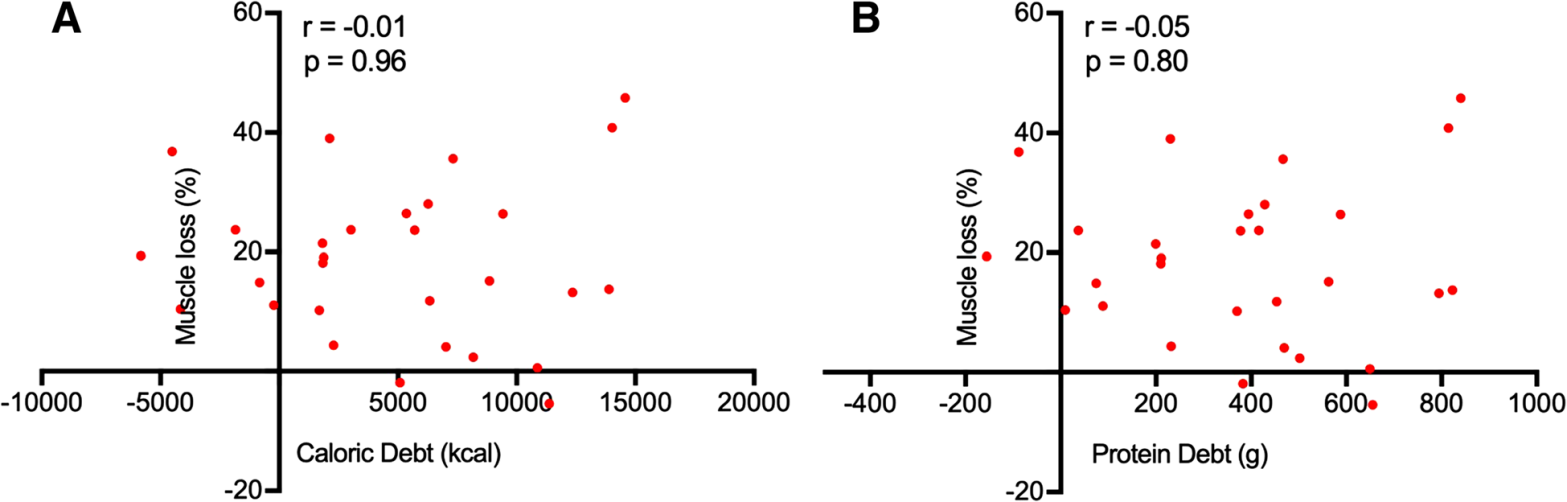
Correlation between muscle loss and nutritional debt during the first week of ICU. **a** Muscle loss and caloric debt expressed in calories. **b** Muscle loss and protein debt. r: Spearman correlation coefficient

During the first week of the ICU stay, daily caloric intake was 14.6 kcal/kg/day [95% CI,7.4; 20.4] and daily protein intake was 0.4 g/kg/day [95% CI, 0.1; 0.7]. Based on international guidelines, the cumulative caloric debt during the first week was 5353 kcal [95% CI, 2808–7119]) and the cumulative protein deficiency over the same period was 443 g [95% CI, 257.8–548.7]. There was no correlation between caloric (*r* = − 0,01; *p* = 0,96) or protein (*r* = − 0,05; *p* = 0,80) debt and the decrease in muscle mass during the first week of ICU stay (Fig. 4).

## Discussion

Bedside ultrasound muscle assessment may be a convenient non-invasive tool to evaluate muscle wasting in the critical care setting. We demonstrated that this technique was reproducible by an individual observer and accurate between different observers. We observed a higher intra and inter-observer reliability when evaluations were made at the two-thirds of the length between the ASIS and the patella, with ICCs considered as “almost perfect agreement” [24]. Patients suffered a significant muscle loss of 16% within the first week of the ICU stay and 24% at D21.

These measurements, completed as a part of standard care, allowed us to closely monitor muscle wasting and to assess its chronology. The percentage of muscle thickness loss reported during the first week of ICU was consistent with previous studies [3]. The ICCs calculated were in the same range compared to those found in the literature regarding healthy volunteers [19], elderly patients [25], stroke patients [26], septic patients [16] and critically ill patients with acute kidney injury [27]. This study differs from previous work in various aspects. First, our patients had high severity scores (expected mortality of 44%), were mechanically ventilated and received aggressive treatments (neuromuscular-blocking agent, vasopressors) which made them highly susceptible to ICU MW, edema and fluid changes. Second, we have chosen to follow muscle wasting during a prolonged window of 21 days. Third, to our knowledge, our study is the first to compare the reliability of two different measurement sites in order to identify the most precise muscle assessment. Finally, we observed the relationship between muscle loss and nutritional intake which constitutes one of the main challenges for our future daily practice [28]. Lowering the incidence of functional disability through nutritional interventions seems very promising.

Insufficient protein intake constitutes a major factor of ICU acquired weakness. In the critical care setting, the recommended daily protein intake fluctuates between 1.2 and 2.0 g/kg/day [22, 23] but this target is rarely reached in current practice [29, 30]. Our cohort reflects those conclusions with a median daily protein intake of 0.4 g/kg/day and only 7% of patients reaching the recommended protein intake. Furthermore, we did not find a correlation between caloric or protein deficiency and muscle mass change during the first week. A study [29] assessing 119 critically ill patients compared an amino-acid intake of 0.8 g/kg/day or 1.2 g/kg/day delivered by parenteral nutrition. Higher amino-acid provision was associated with significant greater forearm muscle thickness evaluated by ultrasound. Therefore, a future study of the relationship between muscle loss, evaluated by *Rectus femoris* CSA, and daily protein intake would be pertinent.

Our study presents several limitations. Restricting our measurements to *quadriceps femoris* combined thickness and not carrying out *rectus femoris* CSA measurements may constitute the first limit. Indeed, a recent trial published in 2017 [30] highlighted the superiority of muscle CSA as a reliable proxy for muscle strength compared with muscle thickness. The main concern about muscle CSA assessment is the difficulty of obtaining a full image of the *rectus femoris* muscle with conventional high frequency linear probes (6–12 MHz) which have limited depth penetration. A recent study [31] demonstrated the accuracy of using a lower frequency (2–5 MHz) curvilinear ultrasound probe, known to have higher depth penetration with lower resolution, for the evaluation of *Rectus femoris* CSA. Consequently, future studies could easily focus on that technique in order to predict functional outcome in critically ill patients. Furthermore, the fact that both observers were ultrasound trained physicians represents a second limit. Studying the reliability of measurements made by novice students and describing their learning curve could be an interesting extension of our work. The low number of blind dual operator assessments constitutes an additional limitation to this work and the presented results should be confirmed in higher scale studies. Finally, we previously stated that ICU survivors can suffer from long-term functional impairment and quality of life degradation [32], therefore, the limited window of observation in the ICU may not be sufficient. Prolonged muscle loss assessment beyond ICU or hospital stay would shed light on the chronology of muscle wasting and on the possible therapeutic options at our disposal to optimize muscle health.

Finally, ultrasound assessment may constitute a reliable and objective tool to assess frailty and malnutrition in the ICU. Promoting this technique may raise physicians’ attention regarding the prompt onset of muscle loss in the ICU, could encourage them to introduce daily follow-ups in their protocols and motivate them to intervene through nutritional interventions or exercise training. A recent review concerning nutrition monitoring in the ICU insists on the prevention and the early detection of nutritional-related complication through the use of clinical, biological and technical tests. [33] The implementation of such a bundle in the ICU has never been evaluated regarding patient outcome and could provide the basis of further investigation. Furthermore, our work could reinforce the strength of ultrasound assessment as an outcome to predict functional recovery for future randomized controlled trials (RCT) aimed to limit ICU muscle wasting. An article [34] co-written by major ICU nutritional experts recently emphasized the need to use functional outcomes in future RCTs considering that the outcomes commonly studied (mortality, length of stay) may incorrectly reflect the effect of nutritional interventions.

In the last decades, major technological and pharmaceutical progress has helped to considerably lower ICU and in-hospital mortality at the cost of profound and long-term physical disabilities for survivors. Functional recovery and its assessment in critically ill patients should be, from now on, a major focus in future research.

## Conclusions

The assessment by ultrasound of the *Quadriceps femoris* muscle thickness reveals good intra- and inter-observer reliability and constitutes an accurate bedside method to diagnose and monitor acute muscle wasting in critically ill patients. The “two-thirds” site showed better accuracy than the “midpoint site”. In our population, muscle thickness decreased by over 16% within the first week in the ICU and no significant relationship was found with nutritional intake.

## Abbreviations

ASIS: Anterior superior iliac spine
BMI: Body mass index
CSA: Cross-sectional area
ICC: Intra-class correlation coefficient
ICU: Intensive care unit
MW: Muscle wasting
RCT: Randomized controlled trials
SAPS II: Simplified acute physiology score II
US: Ultrasonography
